# The structure of the environment influences the patterns and genetics of local adaptation

**DOI:** 10.1093/evlett/qrae033

**Published:** 2024-08-17

**Authors:** Tom R Booker

**Affiliations:** Department of Forest and Conservation Science, Faculty of Forestry, University of British Columbia, Vancouver, V6T 1Z4, Canada

**Keywords:** local adaptation, population genetics, evolutionary genetics, selection, lodgepole pine

## Abstract

Environmental heterogeneity can lead to spatially varying selection, which can, in turn, lead to local adaptation. Population genetic models have shown that the pattern of environmental variation in space can strongly influence the evolution of local adaptation. In particular, when environmental variation is highly autocorrelated in space local adaptation will more readily evolve. However, there have been few attempts to test this prediction empirically or characterize the consequences it would have for the genetic architecture underlying local adaptation. In this study, I analyze a large-scale provenance trial conducted on lodgepole pine and find suggestive evidence that spatial autocorrelation in environmental variation is related to the strength of local adaptation that has evolved in that species. Motivated by those results, I use simulations to model local adaptation to different spatial patterns of environmental variation. The simulations confirm that local adaptation is expected to increase with the degree of spatial autocorrelation in the selective environment, but also show that highly heterogeneous environments are more likely to exhibit high variation in local adaptation, a result not previously described. I find that the spatial pattern of environmental variation influences the genetic architectures underlying local adaptation. In highly autocorrelated environments, the genetic architecture of local adaptation tends to be composed of high-frequency alleles with small phenotypic effects. In weakly autocorrelated environments, locally adaptive alleles may have larger phenotypic effects but are present at lower frequencies across species’ ranges and experience more evolutionary turnover. Overall, this work emphasizes the profound importance that the spatial pattern of selection can have on the evolution of local adaptation and how spatial autocorrelation should be considered when formulating hypotheses in ecological and genetic studies.

## Introduction

Local adaptation is an important phenomenon in the natural world. Along with phenotypic plasticity, local adaptation can dictate the extent of environmental heterogeneity that a species can tolerate, shape its geographic range (Kirkpatrick & Barton, 1997), and help predict how it will respond to changing environments (Rellstab et al., 2021). In forest trees, for example, local adaptation is widely observed (Leites & Benito Garzón, 2023) and is central to plans for adapting forestry practice in light of climate change (O’Neill & Gómez-Pineda, 2021; Ying & Yanchuk, 2006). Local adaptation can be defined as a kind of genotype-by-environment interaction for fitness, where individuals have higher chances of survival and/or reproduction when they are reared at home as opposed to away, though several other definitions are used in the literature (Blanquart et al., 2013; Kawecki & Ebert, 2004). Under this definition, local adaptation is a property of a particular population at a particular point in time rather than a property of a species as a whole. For example, populations at range edges are often expected to be maladapted to their conditions, while populations in the core of a range may be well adapted (Angert et al., 2020). Locally adapted populations may harbor genetic variation that could help buffer susceptible ones against the detrimental effects of climate change (Aitken & Whitlock, 2013), which are already wreaking havoc on important species around the world (Hartmann et al., 2022). A deep understanding of local adaptation, the agents that have given rise to it and the genetics that underpin this phenomenon is thus important for our understanding of biodiversity and for species management and conservation in the Anthropocene (Aitken & Whitlock, 2013; Exposito-Alonso, 2023; Wadgymar et al., 2022).

The ultimate cause of local adaptation is variation in the environment. Whether it is biotic (e.g., disease/parasite prevalence or intraspecific competition) or abiotic (e.g., climate, geology or photoperiod), variation in the environment may lead to spatially varying selection pressures where phenotypic optima differ over a landscape. Such variation in selection across space has been well documented (e.g., Siepielski et al., 2013). For example, in *Erigeron annuus* genotype-by-environment interactions for fitness linked to factors such as soil chemistry and biotic interactions have been shown to vary over distances as short as 10 cm (Stratton, 1994). In any given species there are, of course, myriad aspects of the environment that could conceivably induce spatially varying selection. However, that a particular aspect of environmental variation can induce spatially varying selection is not a guarantee that it will have led to local adaptation. A recent review by Wadgymar et al. (2022) highlighted a critical gap in our knowledge of local adaptation—that the aspects of environmental variation that have given rise to it (what they term the “agents of selection”) are unknown in most cases. In the absence of experimental evidence many studies have assumed that various climatic measures recorded in databases such as WorldClim correspond to agents of selection and, for example, use these to search for the genetic basis of local adaptation (Lasky et al., 2023).

It has long been recognized that the spatial pattern of environmental heterogeneity will influence the evolution of local adaptation (Antonovics, 1971; Antonovics & Bradshaw, 1970; Forester et al., 2016; Hadfield, 2016; Levins, 1966; Schiffers et al., 2014). Population genetic studies have revealed the reasons for this. The two most important factors that influence the evolution of local adaptation are the strength of selection and rates of gene flow. The strength of natural selection is of foremost importance because larger fitness consequences for deviating from the optimal phenotype in a particular location can potentially lead to greater evolutionary change (Falconer & MacKay, 1995). The rate of gene flow is important because migration into a region experiencing idiosyncratic selection can overwhelm that selection, preventing regional trait differences (i.e., local adaptation) from accumulating (Nagylaki, 1975; Slatkin, 1978; Wright, 1931; Yeaman, 2015). Indeed, whether a locally adaptive allele is maintained in a region depends on the ratio of gene flow from dissimilar environments (*m*) to the strength of selection (*s*), *m/s* (Slatkin, 1978; Wright, 1931; Yeaman, 2015). While those results were derived for models of discrete populations, similar results have been obtained for continuous space models (Barton, 1999; Lenormand, 2002). In the case of polygenic selection, the tension between selection and migration is also expected to determine how quantitative traits vary over space (Barton, 1999; Slatkin, 1978). Selected phenotypes may closely match local optima if differences in optima between locations exchanging migrants are small (Barton, 1999; Slatkin, 1978). Of course, the scale over which the environment varies and the scale over which populations disperse are critically important parameters (Slatkin, 1973). In natural systems, populations may inhabit large spatial ranges that encompass complex patterns of environmental variation where the ratio of selection to migration will vary across the landscape. The way that the environment varies over space is, therefore, expected to influence the evolution of local adaptation.

With respect to the evolution of local adaptation, previous studies have described the structure of the environment in terms of spatial autocorrelation (Hadfield, 2016; Urban, 2011). Spatial autocorrelation describes the similarity of observations from nearby locations and can be quantified, for example, using Moran’s I (Moran, 1950). When the environment that gives rise to spatially varying selection across a species’ range exhibits high spatial autocorrelation, selection pressures may be similar over large areas and changes in environment over space will tend to be gradual. On the other hand, when the environment exhibits weak autocorrelation, regions experiencing idiosyncratic selection will be comparatively small and selection pressures may change rapidly over space. Of course, other factors such as variation in population density, the magnitude of environmental variation and the scale of dispersal will also affect the outcomes of spatially varying selection. All else being equal, however, a species with restricted migration will tend to evolve the strongest local adaptation if agents of selection exhibit high levels of spatial autocorrelation across the species’ range (Hadfield, 2016). There is empirical evidence that spatial autocorrelation in patterns of biotic interactions can explain a substantial proportion of variation in trait differentiation among populations (Urban, 2011). However, empirical evidence that patterns of local adaptation coincide with spatially autocorrelated features of the environment is lacking (Urban, 2011).

Despite strong theoretical predictions about the evolution of local adaptation when the agents of selection are autocorrelated in space, the genetics of local adaptation in such cases are not well characterized. In simple two-patch models, the relative balance of selection and migration influences the number of alleles underlying local adaptation, their effect sizes and rates of allelic turnover (Yeaman & Whitlock, 2011). In continuous, linearly varying landscapes, frequencies of alleles contributing to locally adaptive traits are expected to vary in relation to their effect sizes and the proximity of local trait means to phenotypic optima (Polechová & Barton, 2015). Traits underlying local adaptation may be highly polygenic (Yeaman, 2022), where the phenotypic effects of individual mutations vary across the genome. The rate of range expansion across environmental gradients is expected to be negatively related to the steepness of those gradients and can be influenced by the genetic architectures of selected traits (Gilbert & Whitlock, 2017; Schiffers et al., 2014). However, previous models have not examined the patterns of genetic variation underlying local adaptation that evolve when complex environmental variation causes the relative balance of selection and migration to vary across space. It is, therefore, unclear how genetic architectures of local adaptation will vary depending on the spatial pattern of environmental variation across species’ ranges.

In this study, I examine how the patterns of local adaptation and the genetics underlying it are affected by the spatial pattern of environmental variation. Following previous studies, I cast spatial patterns of environmental variation across a species’ range in terms of spatial autocorrelation. I use empirical data from a large-scale experiment in lodgepole pine and find evidence that spatial autocorrelation in patterns of climatic/environmental variation are related to local adaptation in a natural system. I then build a simulation model of spatially varying selection to investigate how local adaptation and its genetic bases can be influenced by the pattern of environmental variation. I examine the genetic architecture of local adaptation and its evolution as it relates to varying degrees of spatial autocorrelation in the environment. Overall, I show that spatial autocorrelation in the environment can have a profound impact on the patterns and genetics of local adaptation and discuss implications from this work for the study of natural populations.

## Results

### Local adaptation, environmental autocorrelation, provenance trials, and lodgepole pine

**Figure 1.**
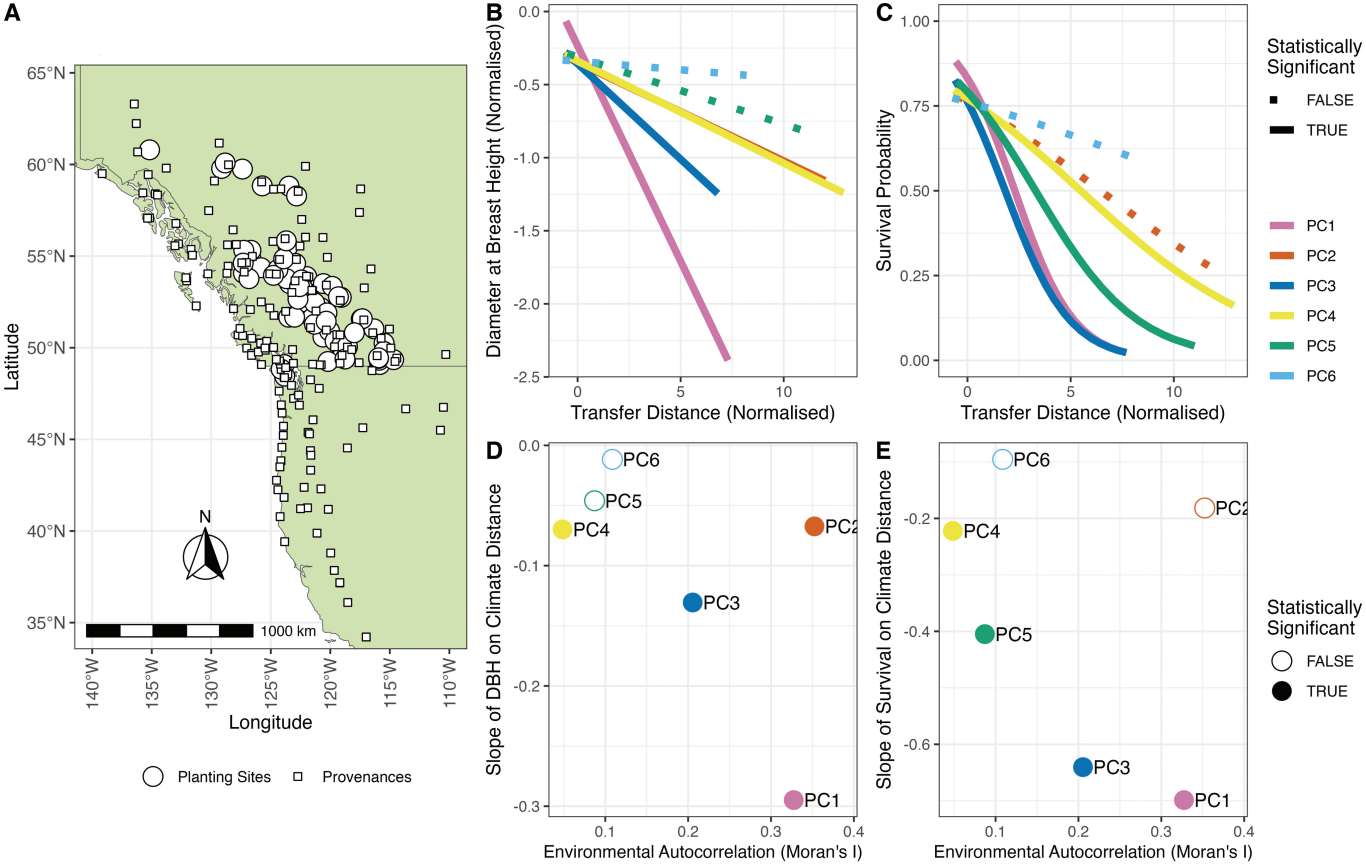
Analysis of local adaptation in lodgepole pine from the Illingworth provenance trial. (A) The map of provenances and planting sites in the Illingworth trials across the Northwest of North America. (B Fitted relationships between tree diameter at breast height (DBH) and transfer distance for 8 principal components. (C) Survival probability as a function of transfer function for 8 environmental principal components. (D) Linear regression coefficients for the relationships shown in B compared to degree of autocorrelation in the environment. (E) Logistic regression coefficients for the relationships shown in C compared to the degree of spatial autocorrelation in the environment. Statistical significance was assessed at *ɑ* = 0.05 after correcting for multiple comparisons using the Dunn-Šidak method.

Local adaptation is widely observed in forest trees (Leites & Benito Garzón, 2023) and, in some regions, this is central to plans to adapt forestry practice in light of climate change (O’Neill & Gómez-Pineda, 2021; Ying & Yanchuk, 2006). Provenance trials have been used for almost 200 years in forest trees and involve planting multiple populations of a species in numerous common gardens to assess how “transfer distance,” the distance between home and the common garden, affects productivity (reviewed by Wadgymar et al., 2022). By regressing fitness proxies on transfer distance, data from provenance trials can be used to identify and measure the strength of local adaptation (Supplementary Figure S1). The Illingworth trial is an exceptionally large provenance trial established by the Ministry of Forestry in British Columbia, Canada in the 1970s to establish seed-transfer guidelines for the lodgepole pine (*Pinus contorta*) (Illingworth, 1978). Seeds were collected from 140 provenances from Northwestern North America and seedlings were planted in a set of 62 sites distributed across British Columbia (Figure 1A). Phenotypic data has been recorded for around 60,000 individual trees since the Illingworth trial began and previous studies have used these data to demonstrate clear local adaptation in lodgepole pine (Mahony et al., 2020; Wang et al., 2006). Given the geographic breadth of the Illingworth trial (Figure 1A), it represents a suitable dataset to test the prediction that the spatial pattern of environmental variation influences the evolution of local adaptation.

Lodgepole pine exhibits the strongest signals of local adaptation when climatic/environmental variation is highly spatially autocorrelated (Figure 1). I analyzed data from the Illingworth trial using a mixed-modeling approach (see *Methods*). Different aspects of climatic variation across the sites in the Illingworth trial are highly intercorrelated (Supplementary Figure S2), so I used principal components analysis to identify independent axes of climatic/environmental variation in the dataset. Across all locations in the Illingworth trial, there were 6 principal components (PCs) that explained a combined total of 95% of climatic/environmental variation (Supplementary Figure S2A). I regressed phenotypes (either tree diameter at breast height or survival) on transfer distance between planting site and provenances in PC-space (Figure 1B, C), modeling provenance and planting sites as random effects (see *Methods* for details). Among the aspects of climatic variation that had significant slopes of diameter or survival on environmental distance, the steepest slopes were found for the PC with the greatest degree of spatial autocorrelation, as measured using Moran’s I (Figure 1D, E). The PC in question (PC1) captures climatic/environmental differences between coastal and inland locations (Supplementary Figure S3). Overall, there seemed to be a negative association between the strength of local adaptation and the degree of spatial autocorrelation in the environment (Figure 1D, E). However, I did not conduct a formal statistical test of the relationship between spatial autocorrelation and local adaptation because only 4 PCs exhibited statistically significant evidence for local adaptation so such an analysis would be underpowered. While this means the results are merely suggestive rather than concrete, they are in line with the prediction that spatial autocorrelation in the abiotic environment predicts the strength of local adaptation in natural populations.

### Simulating local adaptation to spatially heterogeneous environments

**Figure 2.**
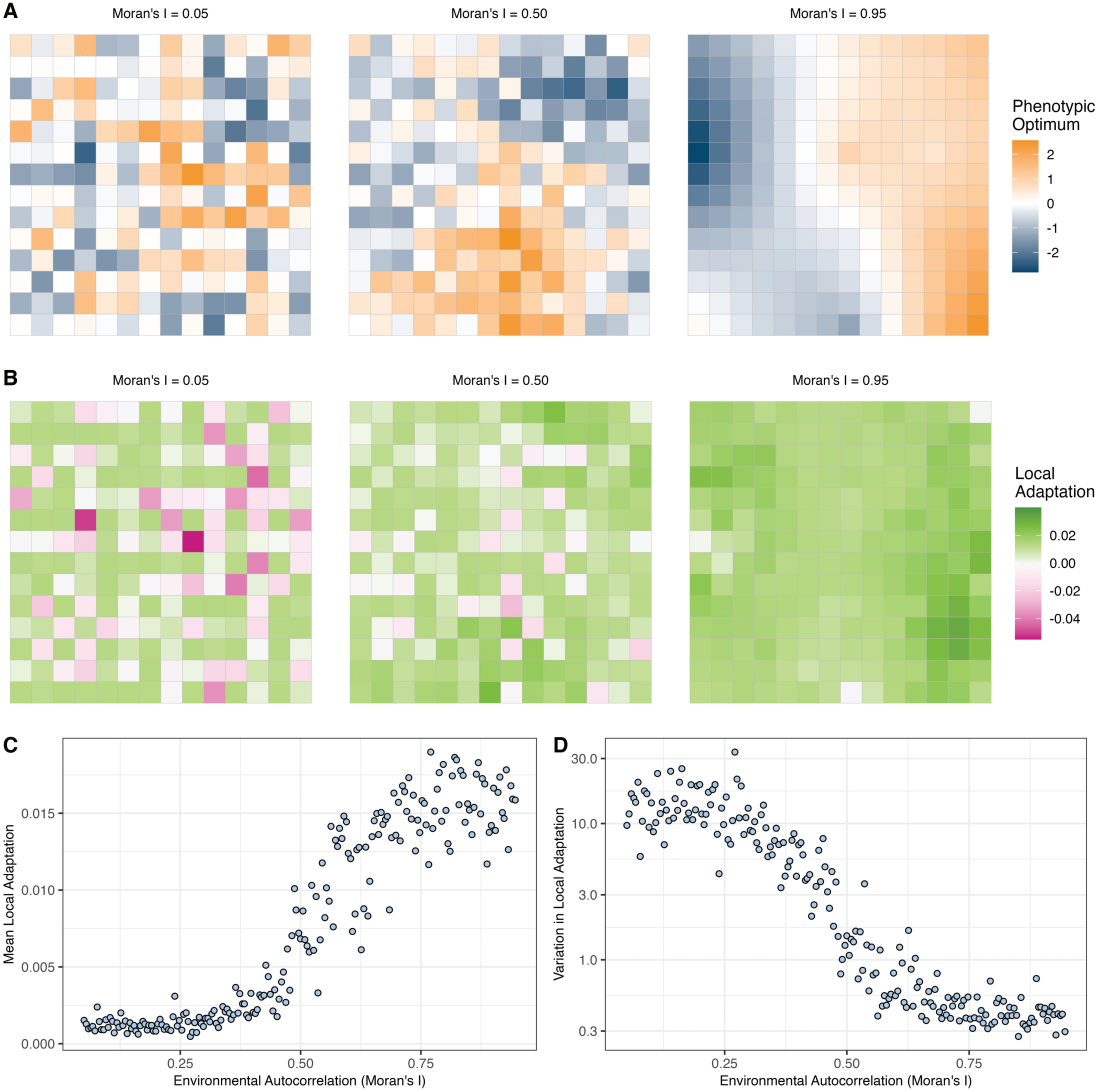
The pattern of environmental heterogeneity influences the outcomes of spatially varying selection. (A) Three examples of environmental heterogeneity with similar distributions of phenotypic optima ranging from low autocorrelation on the left to high autocorrelation on the right. (B) The pattern of local adaptation that evolved on the landscapes shown in panel A. (C) The average local adaptation that arises as a function of Moran’s I across 200 maps of environmental variation. (C) The coefficient of variation for local adaptation across the 200 maps. The simulation results shown are for cases with mean *F*_ST_ ~ 2% and moderate stabilizing selection.

To develop our understanding that the effects of the spatial pattern of environmental variation can have on patterns of local adaptation, I constructed a simulation model of spatially varying selection. I used forward-in-time population genetic simulations in SLiM (4.1; Haller & Messer, 2023) modeling a 2-dimensional stepping-stone metapopulation of 196 demes (i.e., a 14 × 14 grid). Migration was restricted to adjacent demes with rates of gene flow that resulted in pronounced population structure with clear isolation-by-distance (Supplementary Figure S4). I implemented a model of spatially varying stabilizing selection where each deme () had a particular phenotypic optimum (). I constructed a set of 200 maps of normally distributed environmental variation that varied in the degree of spatial autocorrelation (three examples are shown in Figure 2A) to specify phenotypic optima. I quantified spatial autocorrelation in the environment using Moran’s I, which varied from 0.05 (weak autocorrelation) to 0.95 (strong autocorrelation) across the maps I constructed. For further details and justification of parameter choices, see *Methods*.

Across the simulated populations, the effects of environmental structure on patterns of local adaptation that evolved were profound. I measured local adaptation in simulated populations by comparing an individual’s fitness at “home” versus “away” following the method outlined by (Blanquart et al., 2013). As expected, the mean local adaptation across populations ($\bar{LA}$) increased with the degree of spatial autocorrelation in the environment (Figure 2B, C), consistent with Hadfield (2016). This result held over various rates of gene flow and strengths of stabilizing selection (Supplementary Figure S5). Varying the rate of gene flow and/or the strength of selection had an effect on the level of local adaptation that arose in a given case, but a pattern of increasing $\bar{LA}$ with Moran’s I was always observed (Supplementary Figure S5). In cases where simulated populations had multiple traits subject to spatially varying selection, traits corresponding to more highly autocorrelated environment exhibited greater local adaptation (Supplementary Figure S6).

The spatial pattern of environmental variation did not just affect the average level of local adaptation, though, it also had a large influence on the variation in local adaptation across the landscape (Figure 2B). The coefficient of variation in local adaptation across the landscape decreased rapidly with increasing autocorrelation (Figure 2D; Supplementary Figure S5B). When environmental variation was weakly autocorrelated, was as much as 30× higher than for more highly autocorrelated environments (Figure 2D). Variation in the degree of local adaptation across a species range is understudied in the population genetics literature but has important implications (see *Discussion*).

At a finer scale, environmental variation in the immediate vicinity of a particular deme predicted its level of local adaptation and genetic variation, as predicted by theory (Barton, 1999; Guillaume & Whitlock, 2007; Slatkin, 1978). Demes that were surrounded by populations with highly similar phenotypic optima evolved greater local adaptation than demes bordering more dissimilar environments (Supplementary Figure S7A). This was particularly evident when the overall landscape was weakly autocorrelated (Supplementary Figure S7A), presumably because in highly autocorrelated landscapes most demes are surrounded by similar environments. Additive genetic variance (*V*_*A*_) for the trait under selection was highest in demes surrounded by dissimilar environments (Supplementary Figure S7B), suggesting that gene flow among locally divergent populations has an effect of increasing genetic variability. Such a positive correlation between *V*_*A*_ and local environmental heterogeneity has been reported in lodgepole pine (Yeaman & Jarvis, 2006). The magnitude of a species’ response to selection on a trait is expected to be proportional to *V*_*A*_ (Falconer & MacKay, 1995), thus the historical pattern of environmental variation that local adaptation evolves under likely influences a species’ response to future environmental change.

### Environmental structure and the genetic architecture of local adaptation

The results so far demonstrate that the structure of the environment can have a clear impact on the patterns of local adaptation that evolve, but does it influence the genetic basis of that adaptation? Under a model of spatially varying stabilizing selection, each polymorphism that affects the phenotypes under selection will influence local adaptation, but the extent of this will depend on its effect size, where it is present and its allele frequencies. For each polymorphism in a simulation, I quantified the contribution it makes to mean local adaptation ($\bar{LA}$) as follows. I shuffled the presence/absence of a particular polymorphism across the landscape, effectively erasing its contribution to local adaptation. Average local adaptation was then recalculated without the contribution of the focal polymorphism ($\bar{LA_{i}}$). The relative contribution of the focal polymorphism to mean local adaptation across the landscape was then calculated as $LA_{Rel,l}=\left(1-\bar{LA_{i}}/\bar{LA}\right)$. For example, a polymorphism with $LA_{Rel}\approx 1.0$ would be the basis of all local adaptation, while one with $LA_{Rel}\approx 0.0$ would have no effect. Note, $LA_{Rel}$ is not strictly a proportion (see *Methods* for details).

**Figure 3.**
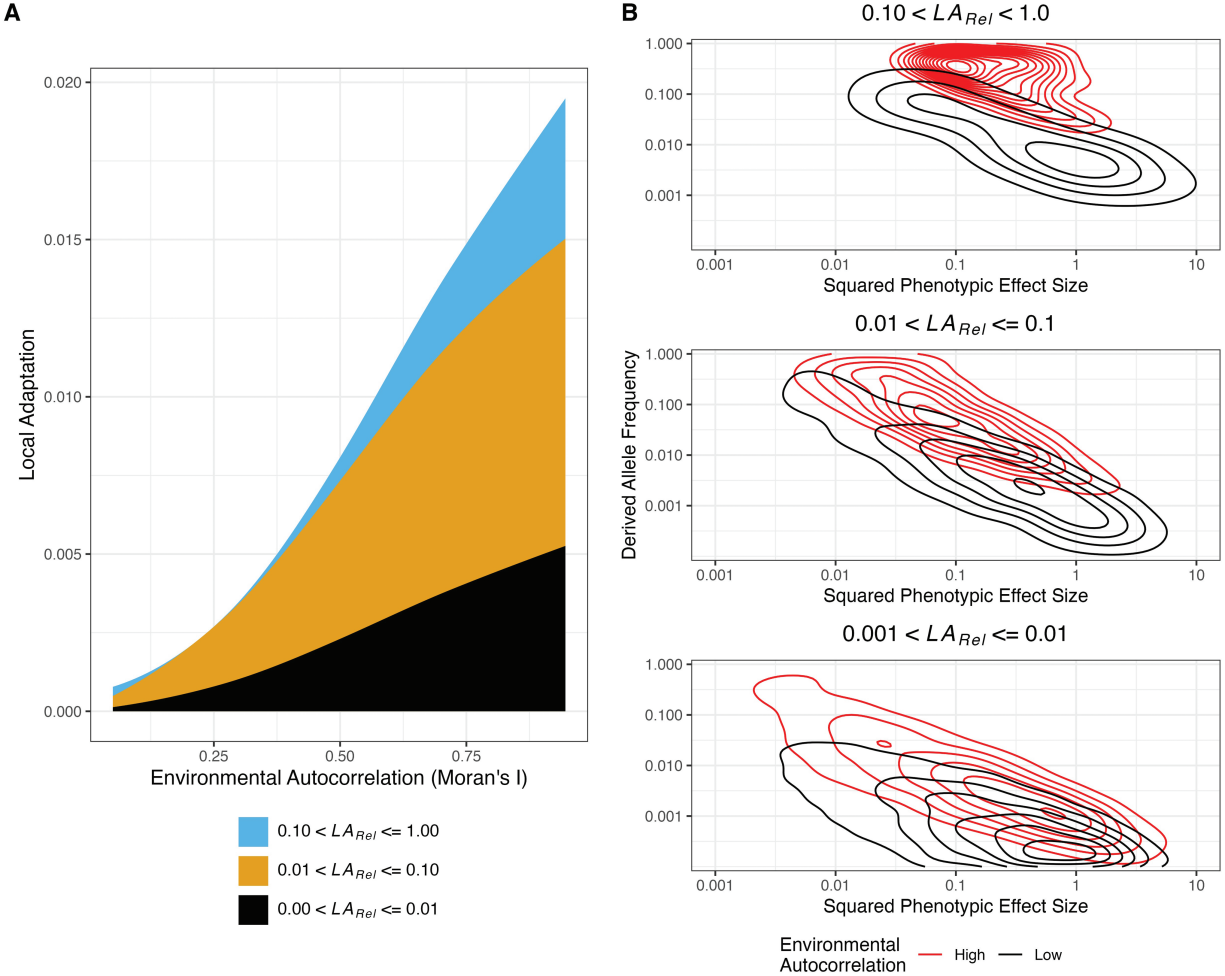
The genetic architecture of local adaptation is influenced by the structure of the environment. (A) The proportion of total local adaptation explained by alleles that individually explain different amounts of local adaptation (LA_Rel_) varies as a function of environmental autocorrelation. Lines represent LOESS curves fit with a span parameter of 1.5. (B) The mean allele frequencies compared to the squared effect sizes of polymorphisms that underly local adaptation differ depending on the pattern of the environment. The contour lines indicate regions with high densities of points. High autocorrelation refers to data from maps with the 50 highest values of Moran’s I. Low autocorrelation refers to data from maps with the 50 lowest values of Moran’s I. Results in both panels come from simulations with *F*_ST_ = 0.02 and moderate stabilizing selection.

The distribution of locally adaptive effects varied in relation to the pattern of environmental variation (Figure 3A; Supplementary Figure S8). In environments exhibiting weak autocorrelation, polymorphisms that individually made a large contribution to local adaptation across the species’ range ($LA_{Rel}>0.10$) were largely absent and polymorphisms that made intermediate ($0.01<LA_{Rel}<0.10$) and small contributions ($LA_{Rel}<0.01$) explained most of the local adaptation that evolved (Figure 3A). In environments that were more highly autocorrelated, polymorphisms with $LA_{Rel}>0.10$ made a substantial contribution to local adaptation alongside those with intermediate and small effects, particularly under strong stabilizing selection (Supplementary Figure S8). These general patterns held over different levels of gene flow (Supplementary Figure S8).

Patterns of genetic variation underlying locally adaptive polymorphisms varied depending on the degree of spatial autocorrelation in the environment. In general, locally adaptive polymorphisms with similar LA_Rel_ tended to have smaller phenotypic effects but larger allele frequencies in highly versus weakly autocorrelated environments (Figure 3B). In highly autocorrelated environments, alleles may readily spread among populations facing similar environmental challenges. However, when alleles with large phenotypic effects spread among neighboring demes, they may cause individuals to overshoot their respective phenotypic optima, so the alleles that are maintained may tend to have smaller phenotypic effects. In weakly autocorrelated environments, on the other hand, genes flowing from one location to another have a much greater chance of encountering highly divergent environments, preventing locally adaptive alleles from spreading across wide regions. In such cases, phenotypic effects may need to be large for locally adaptive mutations to withstand the swamping effects of gene flow. These general patterns were observed with both strong and moderate selection (Figure 3B; Supplementary Figure S9A) as well as over varying levels of gene flow (Supplementary Figure S9A). Indeed, the patterns of allele frequency versus phenotypic effect still held when looking at absolute effects on local adaptation, though they were much less pronounced (Supplementary Figure S9B).

There are numerous factors that may interact with the pattern of spatially varying selection to shape the genetics of local adaptation that I did not explore here. Varying the degree of genetic redundancy in relevant traits, the distribution of phenotypic effect sizes, mutation rates and patterns of dispersal may all influence the genetics of local adaptation (Láruson et al., 2020; Yeaman, 2013; Yeaman & Whitlock, 2011). Follow-up studies looking at how such factors influence the genetic basis of local adaptation in differently structured environments are needed. Nevertheless, the results from the simulations should provide researchers seeking to characterize the genetic basis of local adaptation with useful intuition.

### The evolution of local adaptation: maladaptation and allelic turnover

**Figure 4.**
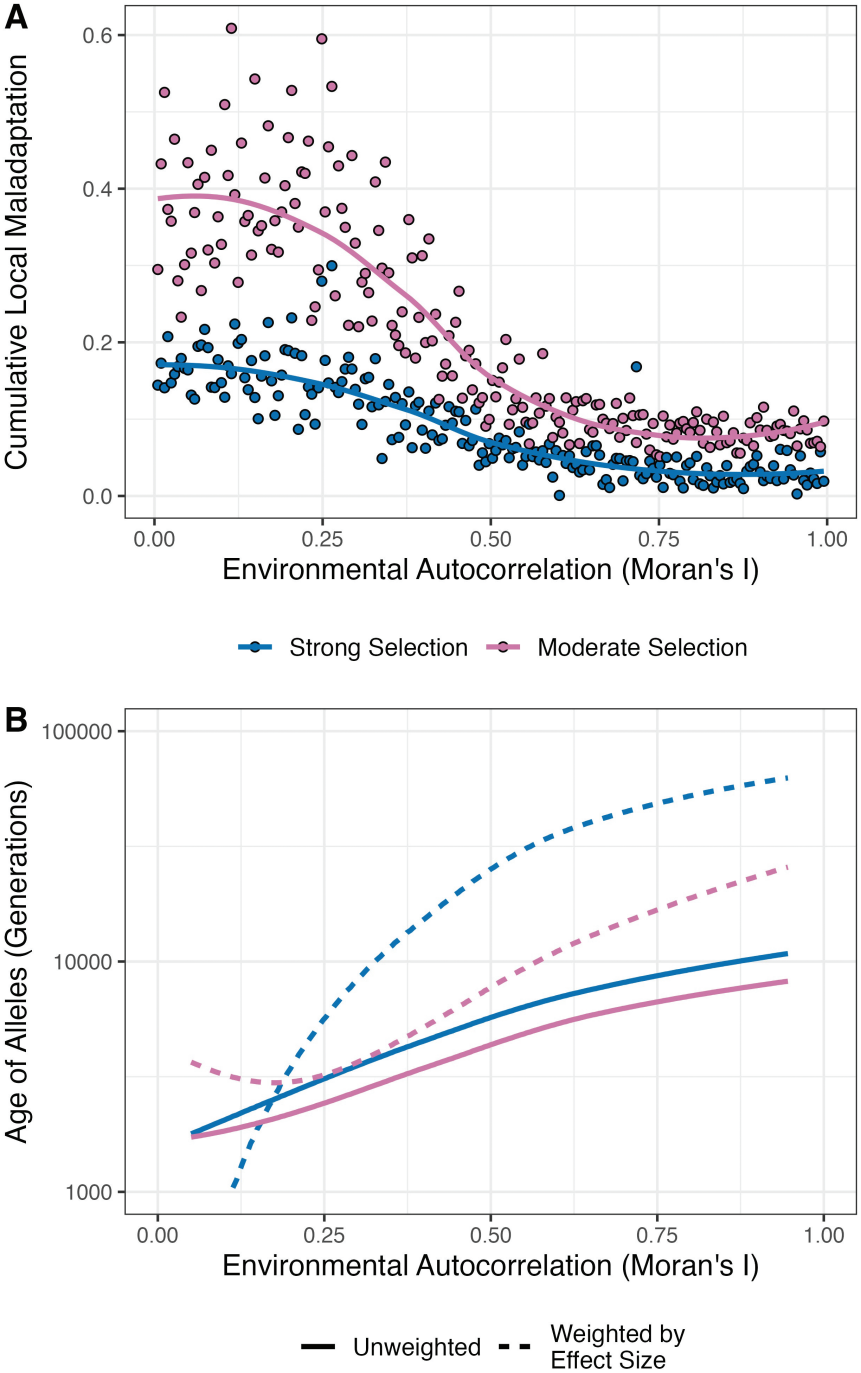
Species-wide maladaptation and the age of locally adaptive alleles are influenced by the pattern of environmental variation. (A) Cumulative local maladaptation, the summed effects of all polymorphism that have a net negative effect on local adaptation across a simulated species’ range, decreases with increasing autocorrelation. Points represent individual simulations and lines represent LOESS curves fit with a span parameter of 1.5. (B) Alleles underlying local adaptation tend to be older when the environment is more highly autocorrelated. Lines represent LOESS curves fit to the data treating all polymorphisms equally, or by giving higher weight to polymorphisms with greater effect size.

Weakly autocorrelated landscapes exhibited high variation in local adaptation across the simulated species’ range (Figure 2). In heterogeneous environments certain polymorphisms may have a net effect of reducing local adaptation across a species’ range. All populations will harbor such locally maladaptive alleles, because any new mutation that increases distance between an individual’s phenotype and the local optimum will reduce local adaptation even if only by a miniscule amount. The cumulative effects of locally adaptive polymorphisms were smaller in weakly heterogeneous environments (Figure 3), but much of the variation in local adaptation across the range in such cases likely stems from maladaptation. By summing the effects of all polymorphisms with $LA_{Rel}<0$ across a simulation, I obtained a measure of the cumulative local maladaptation across a meta-population. Note that the cumulative local maladaptation is analogous to “migration load.” All simulations exhibited some degree of maladaptation regardless of the level of autocorrelation, but the cumulative effects of maladaptive alleles were always higher under weakly versus highly autocorrelated environments (Figure 4A; Supplementary Figure S10). Increasing the rate of gene flow increased the degree of maladaptation and increasing the strength of selection decreased it (Supplementary Figure S10).

Locally adaptive alleles can leave footprints in patterns of genetic variation in regions surrounding selected sites via hitchhiking (Sakamoto & Innan, 2019). Regardless of the pattern of environmental variation in a simulation, levels of local adaptation had been maintained at a steady state for many generations before they were sampled (Supplementary Figure S11). However, the average age of alleles underlying local adaptation increased with increasing autocorrelation in the environment (Figure 4B). Furthermore, weighing the average allele age within a simulation by effect size, the increase in allele age with spatial autocorrelation was even more pronounced (Figure 4B; Supplementary Figure S10). Thus, large effect alleles, in particular, are maintained for longer times in more highly autocorrelated landscapes. Taken together, these results demonstrate that the rate of allelic turnover is greater for more weakly autocorrelated environments. Since the hitchhiking footprints surrounding locally adaptive alleles become more pronounced with age (Sakamoto & Innan, 2019), locally adaptive alleles may tend to leave larger genomic signals in more highly autocorrelated environments.

## Discussion

The analysis of the Illingworth trial (Figure 1) and the simulations should serve to emphasize the importance of spatial autocorrelation in the climate/environment as an important predictor of local adaptation in natural populations. While the effect of spatial autocorrelation on the mean local adaptation is a long-standing expectation of evolutionary biology (Hadfield, 2016; Levins, 1966), the effect of spatial autocorrelation on the variation in local adaptation has not previously been described. Patterns of spatial autocorrelation in the environment can lead to testable hypotheses about the strength of local adaptation that would be hard to formulate based on unrealistic population genetic models such as two-patch or island models. Unlike dispersal or the strength of selection, which are hard to quantify, spatial autocorrelation in the environment is readily measurable.

However, that local adaptation is predicted to be stronger with increasing autocorrelation in the environment does not imply that strong local adaptation cannot arise in highly heterogeneous environments. There are numerous examples of local adaptation to environmental heterogeneity that is not smoothly distributed in space. For example, heavy metal concentrations in mine tailings impose selection that is so strong it overwhelms the effects of gene flow (Jain & Bradshaw, 1966). Indeed, the patchy distributions of environmental variation across a landscape, as may be the case for heavy-metal rich soils, may be more or less autocorrelated from the perspective of a given species depending on its dispersal behavior. In my simulations, I matched the granularity of dispersal with that of the environment (Figure 2A). For real species, considering environmental variation at a scale relevant to patterns of dispersal is critical and recent population genetic advances may make estimating migration surfaces or dispersal behavior in natural populations much less time-intensive than previously (Bradburd & Ralph, 2019; Marcus et al., 2021; Smith et al., 2023). Comparing patterns of species dispersal with patterns of variation in environmental variation that is plausibly relevant to selection may help identify the drivers of local adaptation in natural populations.

In recent years, population genetic analyses have been developed to identify parts of a species range that are particularly vulnerable to climate change (i.e. genomic offset; Rellstab et al., 2021). Such methods analyze present-day relationships between allele frequency and the environment to predict how species will fare given predicted patterns of environmental change. At the core of offset analyses is the assumption that populations are well adapted to their conditions. If the environmental variation underpinning local adaptation is highly spatially autocorrelated, neutral population structure may be partially aligned with gradients of selection, which could explain why the use of putatively adaptive genetic markers and randomly chosen markers seem to perform equally well in some offset analyses (Fitzpatrick et al., 2021; Láruson et al., 2022; Lind et al., 2024). If the agents of selection are weakly autocorrelated, there can be a lot of variation in the degree of local adaptation across species’ ranges (Figure 2). Such variation would violate the assumption of homogeneous local adaptation in offset analyses and likely introduce noise into predictions leading to potentially spurious results.

### Environmental structure and the genetic basis of local adaptation

When seeking to characterize the genetic basis of local adaptation, studies comparing different aspects of environmental variation should consider the pattern of such variation when forming their hypotheses and interpreting their results. As in lodgepole pine (Figure 1), species may exhibit local adaptation to multiple axes of environmental variation simultaneously. Many studies have used population genomic data to try and characterize the genetic basis of local adaptation by analyzing genotype–environment associations (GEA; Lasky et al., 2023). However, Forester et al. (2016) showed that landscape structure influences false positive and negative rates of GEA analyses. Other causes of false positives and false negatives in GEA analyses are reviewed by Hoban et al. (2016). By examining the spatial patterns of various bioclimatic variables (e.g., from WorldClim), researchers could form a priori expectations for the kinds of genetic variation they may expect to underly local adaptation. For example, a patchy distribution of environmental variation may cause different populations to adapt to similar conditions via different suites of alleles, though this likely depends on the genetic architecture of the phenotypes under selection (i.e., genetic redundancy; Láruson et al., 2020). More continuously varying agents of selection may give rise to locally adaptive alleles that are widely distributed. Researchers should, thus, be cautious when trying to characterize the genetic basis of local adaptation to multiple aspects of environmental variation that exhibits markedly patterns of spatial autocorrelation.

The relationship between population structure and the structure of the environment likely impacts our ability to identify the genetic basis of local adaptation. It is well established that a pattern of isolation by distance can confound the search for genes involved in local adaptation (Meirmans, 2012). Indeed characterizing the genetic basis of local adaptation when the agents of selection are co-linear or co-autocorrelated with patterns of gene flow, termed “isolation by environment,” is particularly challenging (Wang & Bradburd, 2014). Genome-wide association (GWAS) and GEA methods often treat population structure as a nuisance variable and various approaches are taken to correct for it. This is done for the statistical necessity of establishing a suitable null model (Meirmans, 2012). Inadequate control for even fine-scale population structure has caused some to misidentify local adaptation from GWAS analyses (Berg et al., 2019; Sohail et al., 2019). In the case of GEA, latent factor mixed models (LFMMs) are widely used to correct for population structure (Caye et al., 2019; Frichot et al., 2013). However, the sensitivity of the LFMM method declines with increasing correlation between the environment and major axes of population structure (Lotterhos, 2023). In a recent analysis of genomic data from red spruce, Capblancq et al. (2023) used two GEA methods, one that corrected for population structure and one that did not. By comparing their results to data from provenance trials, they concluded that a large fraction of local adaptation is confounded with neutral population structure and difficult to identify using GEA methods that controlled for population structure (Capblancq et al., 2023). Careful sampling strategies may alter the power of association methods (Capblancq et al., 2023; Lotterhos & Whitlock, 2015; Meirmans, 2015; Wang & Bradburd, 2014), but such strategies require a priori hypotheses about locally adapted traits, the agents of selection (e.g., Kreiner et al., 2022) and/or the genes involved (e.g., Fournier-Level et al., 2011). Overall, it seems that characterizing the genetic basis of local adaptation may be most difficult when selection pressures and population structure are highly co-autocorrelated over space, i.e., the exact cases where local adaptation is expected to be strongest.

### Closing remarks

While it has been a long-standing expectation that the pattern of environmental variation (and particularly spatial autocorrelation) will influence the evolution of local adaptation (Hadfield, 2016; Levins, 1966), the simulation results and analysis of the lodgepole pine data should serve to emphasize how important the spatial pattern of climatic/environmental variation can be. The spatial pattern of environmental variation that a natural population has experienced will have likely shaped the evolution, current patterns and genetic underpinnings of local adaptation.

## Materials and methods

### Simulating spatially varying selection

To explore the effects of landscape structure on the outcomes of spatially varying selection, I constructed set of maps that exhibited varying degrees of spatial autocorrelation. Maps of normally distributed environmental heterogeneity were constructed using the midpoint displacement algorithm as implemented in the *NLMpy* package (Etherington et al., 2015). I simulated a 14 × 14 cell grid (i.e., landscape), specifying the desired level of autocorrelation to achieve a set of 200 maps, spanning the range of Moran’s I values from 0.05 to 0.95 (i.e., Moran’s I varied in increments of 0.0045). Simulated maps were rejected if the mean value across the landscape was <0.4 or >0.6. This ensured that the approximately normal distributions of environmental values across the landscape were roughly equivalent across maps.

Using SLiM v4.1 (Haller & Messer, 2023), I modeled a 2-dimensional stepping-stone meta-populations with 196 demes (i.e., a 14 × 14 grid). Each deme contained 100 diploid individuals for total meta-population size of 19,600. Migration occurred between adjacent demes in the four cardinal directions except for populations at the range edge where migrants only moved back into demes they were connected to. Migration rates were set at 0.07, 0.035, or 0.0175, leading to population-wide neutral *F*_ST_ values of 0.02, 0.05, and 0.10, respectively (Supplementary Figure S1A). Each diploid individual had a 10-Mbp long genome that recombined at a constant rate of *r* = 1 × 10^−7^. When modelling a single trait, mutational effects were distributed as *N*(0,1) and occurred at random along the sequence at a rate of *μ* = 10^−10^, corresponding to a mutational variance of 0.001 for the trait subject to stabilizing selection. When modelling two traits, the mutation rate was the same, but effects were modelled as multivariate normal with means of 0, variances of 1, and covariances of 0 (i.e., mutational effects for the two traits were independent). A diploid individual’s phenotype for a given trait was the additive combination of the effects on that trait for the alleles the individual possessed (i.e., mutations were semidominant).

Spatially varying stabilizing selection was modeled using the maps of environmental heterogeneity to specify the distribution of phenotypic optima across the landscape. An individual’s relative fitness *W*_*i*_ was calculated using the standard expression for Gaussian stabilizing selection (Walsh & Lynch, 2018):


$$\begin{array}{l} W_{i}=\exp\left[-\left(\frac{{\left(\alpha_{i,d}-\theta_{d}\right)}^{2}}{2V_{s}}\right)\right] \end{array}$$ (1)

where *V*_*s*_*i*s the variance of the Gaussian fitness function, α_*i*_ is the phenotype of the *i*th individual in deme *d*, and θ_*d*_ is the phenotypic optimum of deme *d*. When modeling stabilizing selection in cases with two traits, an individual’s fitness was calculated as follows:


$$\begin{array}{l} W_{i}=exp\left[-\left(\frac{{\left(\alpha_{i,1,d}-\theta_{d,1}\right)}^{2}+{\left(\alpha_{i,2,d}-\theta_{d,2}\right)}^{2}}{2V_{s}}\right)/2\right], \end{array}$$ (2)

where α_*i*,1,*d*_ and α_*i*,2,*d*_ are the values for traits 1 and 2 for individual *i* in deme *d*, respectively, and θ_*d*,1_ and θ_*d*,2_ are the phenotypic optima for traits 1 and 2, respectively. In effect, an individual’s relative fitness in this 2-trait model is the average of the marginal finesses for each trait.

To achieve an equilibrium of migration, selection and drift, meta-populations evolved for 100,401 generations. Initially, meta-populations evolved under stabilizing selection with an optimum of 0 in all demes. After 400 generations, the landscape was altered to one of the 200 maps of environmental heterogeneity and kept in that state for a further 100,000 generations. At the end of the simulation, phenotypes of each individual in each deme were recorded as well as the genealogical history of the meta-population stored as a tree-sequence. PySlim, tskit, and msprime packages (Baumdicker et al., 2022; Haller et al., 2019) were used to work with the output tree-sequence files. To calculate Weir and Cockerham’s *F*_ST_, neutral mutations were added to the simulated population using PySlim at a rate of 10^−8^/bp.

### Analyzing simulated data

Local adaptation was quantified for each deme using the “home-versus-away” (HA) method outlined by Blanquart et al. (2013). Specifically, each individual’s fitness was quantified in its home deme and every other possible location on the landscape. The mean local adaptation was calculated in each deme as the mean difference in fitness between home and away conditions across all individuals. For each deme *d* not on the edge of the simulated landscape, I quantified local heterogeneity in the landscape as the mean sum of squares between the focal deme’s environment and that of the four adjacent demes (in the cardinal directions). For each deme, across the *n* polymorphisms that affected the phenotype additive genetic variance for the trait was calculated as $V_{A,d}=\overset{n}{\underset{i=1}{\sum }}2p_{i,d}(1-p_{i,d}){\gamma_{i}}^{2}$, where $p_{i,d}$ is the allele frequency of SNP *i* in deme *d* and γ_*i*_ is the phenotypic effect of SNP *i*.

The contribution of individual SNPs to local adaptation was quantified as follows. For each polymorphism that affected the trait(s) under selection, the presence/absence of the allele in different haplotypes in different demes can be represented as a vector of 1s and 0s. By shuffling this vector, the contribution of this polymorphism to local adaptation is effectively erased, while keeping its contribution to additive genetic variance across the species’ range constant. For polymorphism *l,* I recomputed all phenotypes for all individuals after shuffling allele frequencies and re-quantified local adaptation as $\bar{LA_{i}}$. The relative contribution of the focal polymorphism to local adaptation is calculated as


$$\begin{array}{l} LA_{Rel,l}=\left(1-\frac{\bar{LA_{i}}}{\bar{LA}}\right). \end{array}$$ (3)

Note that $LA_{Rel}$ is not strictly a proportion, as epistasis for fitness that arises in models of stabilizing selection means that $\overset{n}{\underset{i=1}{\sum }}LA_{Rel,i}\neq 1$ for the *n* SNPs that affect phenotypes. Furthermore, alleles that have a net negative effect on local adaptation (i.e., they are locally maladaptive) will have negative LA_Rel_ values. Indeed, the total amount of local maladaptation in a meta-population was calculated as the additive combination of all polymorphisms with negative LA_Rel_.

Provenance trials were conducted on simulated data by sampling a set of 50 “planting sites” and a set of 100 “provenances.” The relative fitness of each provenance was computed in each of the 50 planting sites. The absolute difference in phenotypic optimum for each provenance and each planting site was used as environmental distance. Using *lme4* in R, I fitted a linear mixed model regressing relative fitness on environmental distance with provenance as a random effect, with slopes and intercepts varying across provenances.

I combined results across the 50 simulations with the lowest levels of spatial autocorrelation (weak autocorrelation) and the 50 simulations with the highest autocorrelation (high autocorrelation) and examined the relationship between allele frequency and phenotypic effect sizes for the alleles underlying local adaptation.

### Analysis of data from the Illingworth trial

ClimateNA (Wang et al., 2016) was used to extract climatic data for each location in the Illingworth Trial. Across the locations in the Illingworth trial, many aspects of climatic/environmental variation are highly inter-correlated (Supplementary Figure S11B). Because such inter-correlation would make it difficult to tease apart the effects of individual aspects of climatic/environmental variation on local adaptation, I conducted a principal components analysis (PCA) to separate the variation onto independent axes. I restricted the analysis to the first 6 principal components as these explained 95% of climatic variation. Diameter at breast height (DBM) and tree height exhibit a strong positive correlation (Pearson’s *r* = 0.9), so analyses were restricted solely to DBM. Trees that were dead or dying after 20 years were given a survival score of 0, living trees were scored a 1.

Phenotype and survival data after 20 years for individual trees from the Illingworth trial were analyzed using mixed models. Mean normalized DBH was modelled as a normally distributed variable using the *lme4* package and survival using a generalized linear mixed model with a “logit” link function using the *glmer* package. The normalized Euclidean distance between each individual’s provenance and planting site (i.e., transfer distance) in PC-space was used as a predictor in the model. Provenance, planting site and planting block within sites were included as having random effects on the slope and intercept of the relationship between phenotype and transfer distance.

Moran’s I was calculated for each principal component of climatic variation across provenances (using the *ape* package) incorporating a pairwise Haversine distance matrix as weights in the calculation.

## Supplementary material

Supplementary material is available online at *Evolution Letters*.

qrae033_suppl_Supplementary_Material

## Data Availability

All the code used to perform, analyze, and plot the results of simulations is available at https://github.com/TBooker/LocalAdaptationArchitechture. R scripts to analyze and plot the results of the Illingworth trial data are available at https://github.com/TBooker/LocalAdaptationArchitechture, but the raw data files were used by permission of the BC Ministry of Forestry.
