# Supplementary material for: The structure of the environment influences the patterns and genetics of local adaptation
Source: Evol Lett. 2024 Aug 17;8(6):787–98. doi: 10.1093/evlett/qrae033 (PMC11637683; doi:10.1093/evlett/qrae033)
Supplement: qrae033_suppl_Supplementary_Material [file qrae033_suppl_supplementary_material.pdf]

Supplementary Material

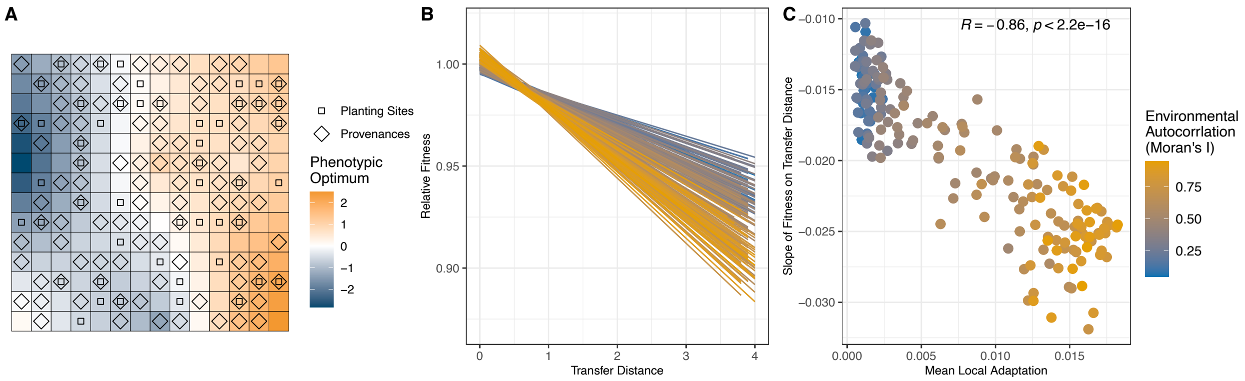

Supplementary Figure 1 Comparing the results from a simulated provenance trial to measures of local adaptation. A) A map of a provenance trial conducted on a simulated population showing the locations of planting sites and provenances. B) Linear regressions of relative fitness on environmental transfer distance for landscapes with differing levels of environmental autocorrelation. C) The slope of relative fitness on transfer distance compared to the mean local adaptation ($\overline{LA}$) across simulated meta-populations. Spearman's ρ and its *p*-value are shown inset in the panel C.

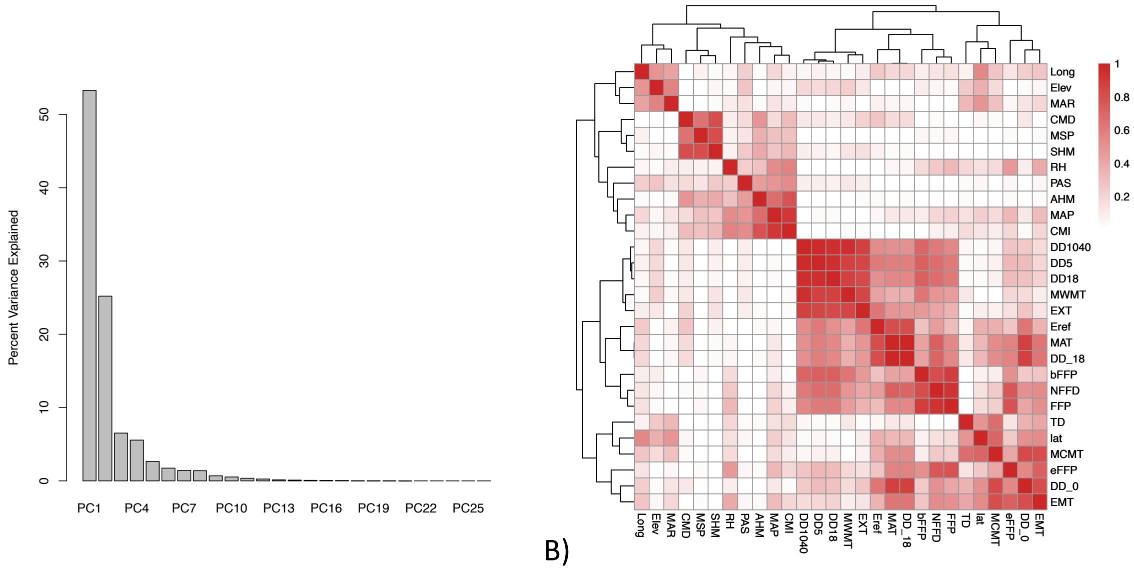

A) B)

Supplementary Figure 2 A) Percent variance explained by the principal component analysis conducted on climatic/environmental variation in the Illingworth trial data. B) The correlation matrix for the 28 climatic/environmental variables for planting sites and provenances in the Illingworth trial. A key to the abbreviations for the 25 annual climatic variables from ClimateBC along can be obtained from https://climatebc.ca/Help2. Additionally, latitude (lat), longitude (Long) and elevation (Elev) are included.

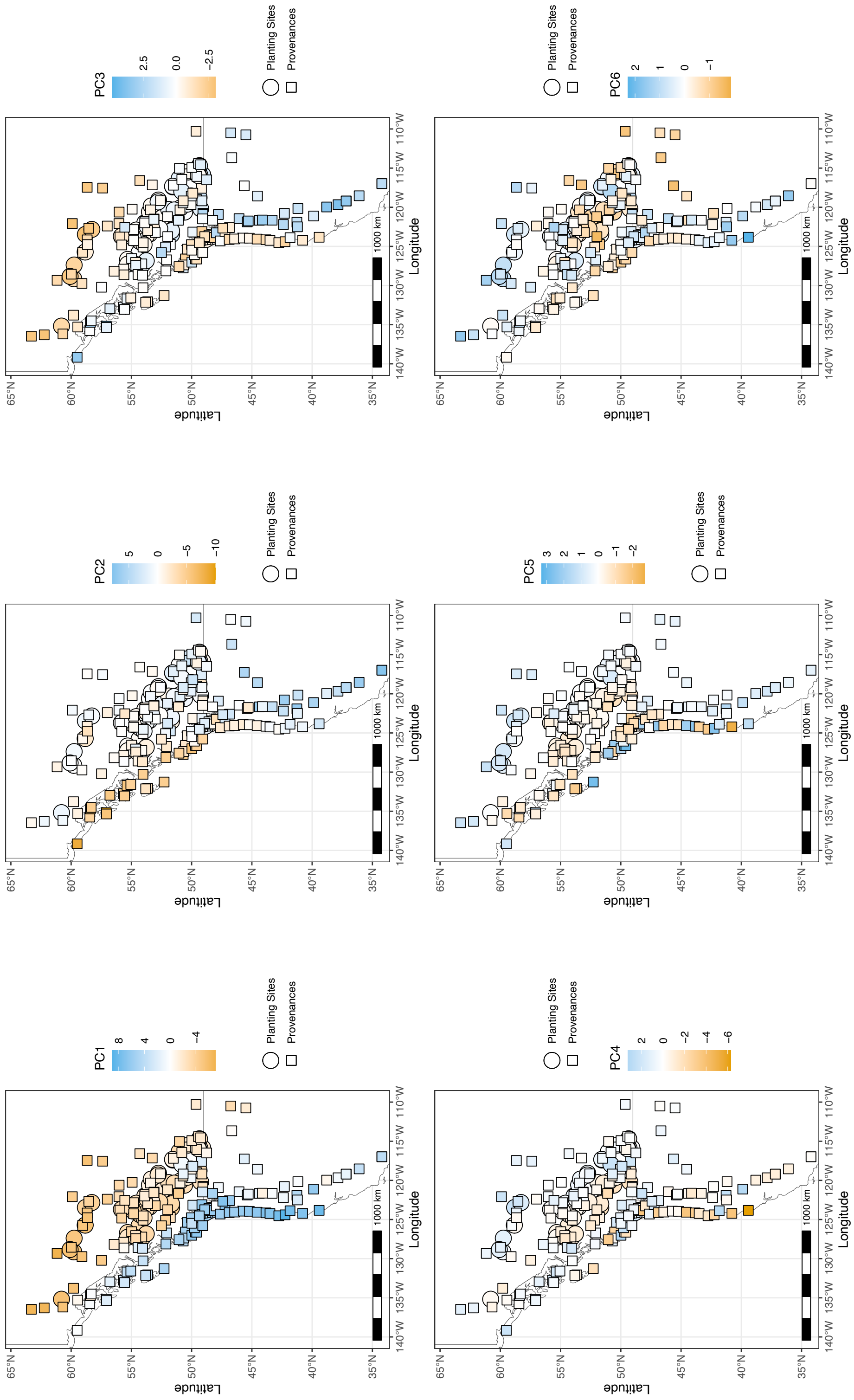

Supplementary Figure 3 The spatial pattern of loadings onto the first 6 principal components of environmental/climatic variation across provenances and planting sites in the Illingworth Trial. The first 6 principal components explained a total of 95% of the variation in the data.

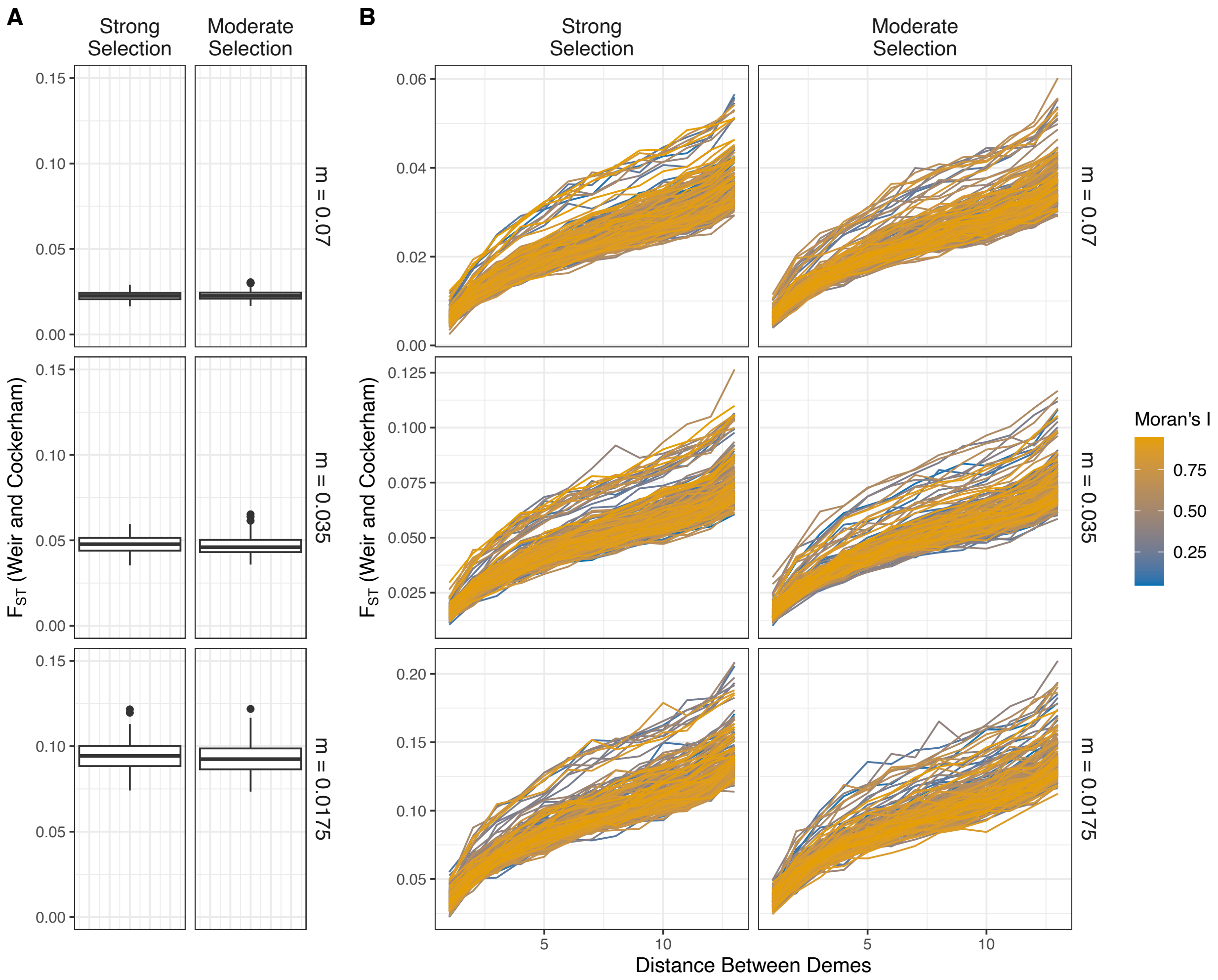

Supplementary Figure 4 Overall F_{ST} (panel A) and isolation by distance (panel B) in simulated populations. Note the varying y-axes in panel B. In the main text, F_{ST} is used to refer to the panels of individual graphs. In panel A values from 200 independent simulations were used to construct the boxplot and in panel B individual simulations are shown as lines. Weir and Cockerham's method for calculating F_{ST}, as implemented in the *sci-kit-allel* Python package*,* was used.

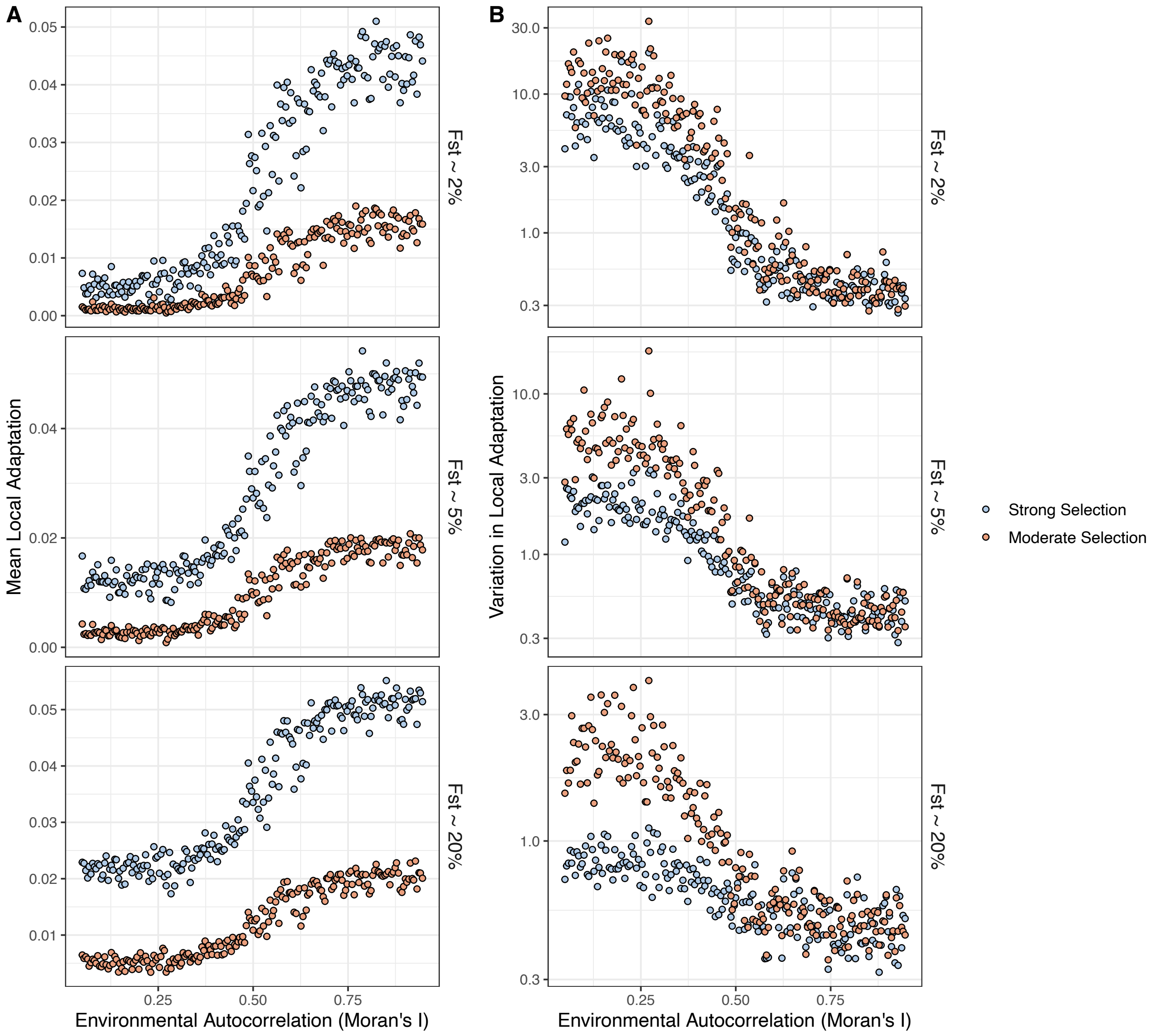

Supplementary Figure 5 The average extent of local adaptation (panel A) and coefficient of variation in local adaptation (panel B) as a function of spatial autocorrelation in the environment from simulated datasets. The upper cell of each column is included in Figure 1 of the main text.

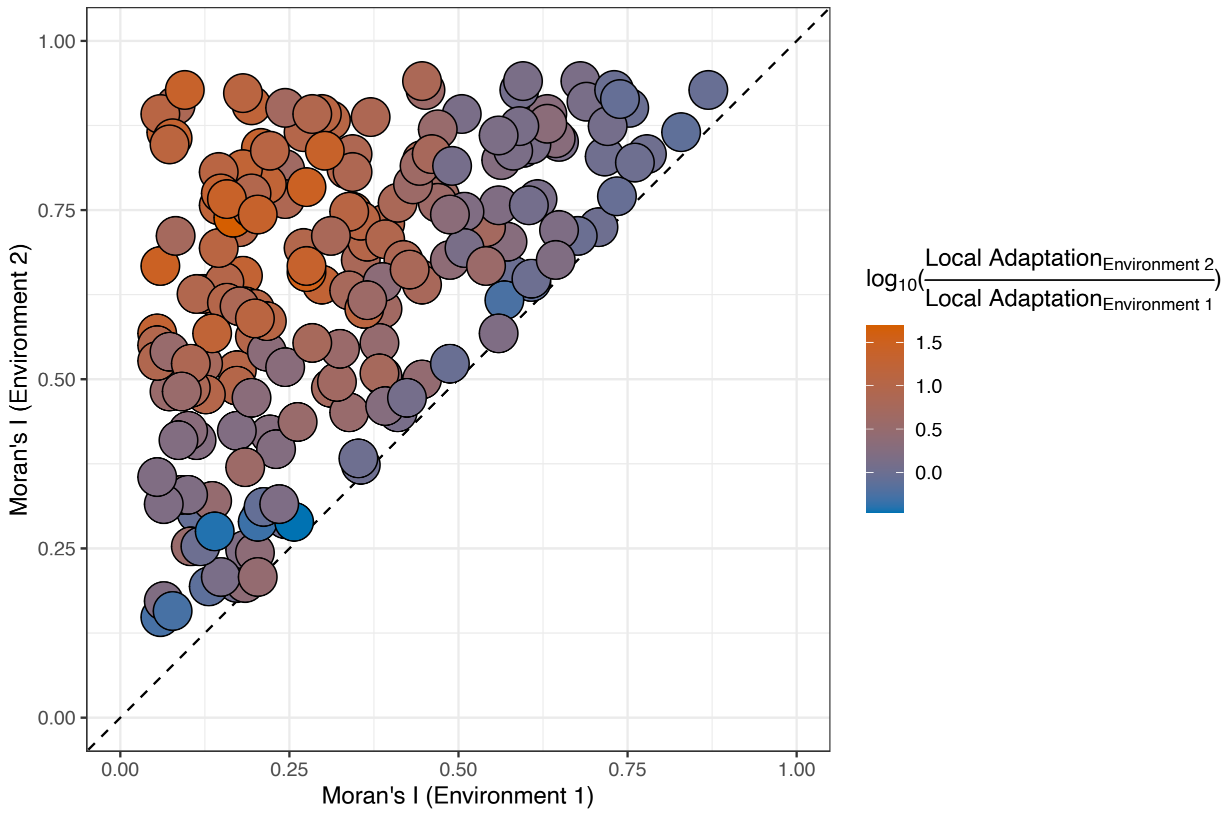

Supplementary Figure 6 Comparison of local adaptation that evolves for two traits subject to spatially varying selection. Selection on each trait was dictated by distinct maps of environmental variation/phenotypic optima. The environment that exhibited the greater degree of spatial autocorrelation (as measured by Moran's I) was designated "Environment 2". The 1:1 line is shown for reference.

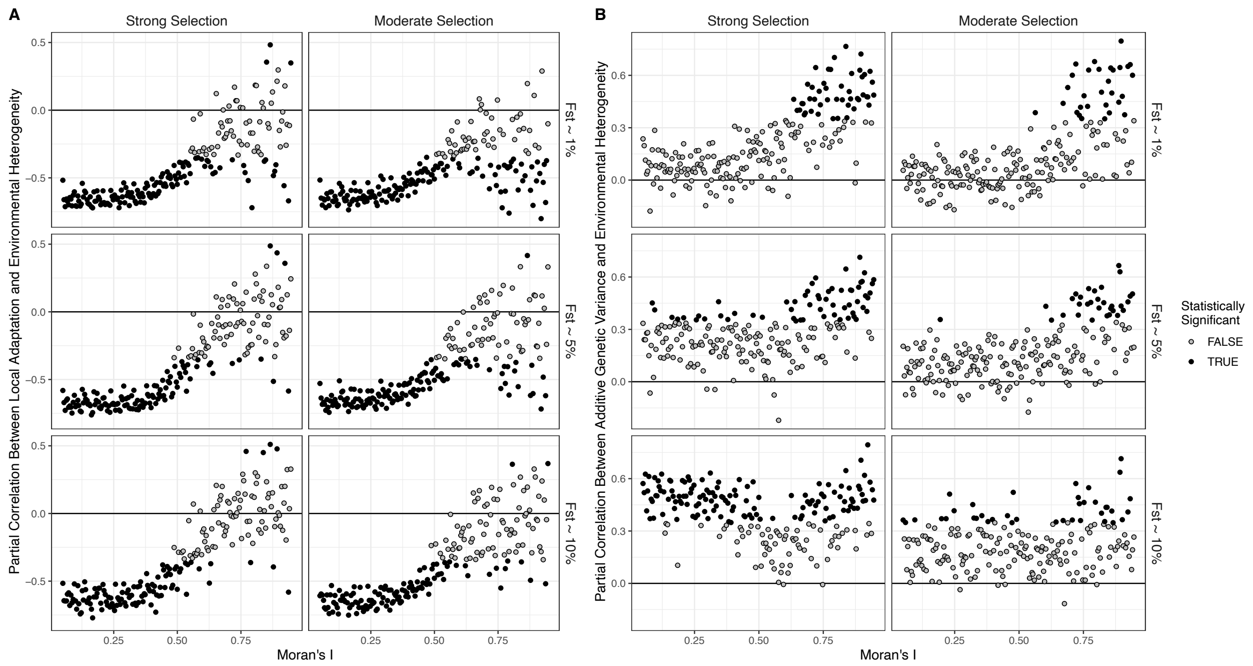

Supplementary Figure 7 The effect of spatial autocorrelation in the environment on the correlations of local environmental heterogeneity with local adaptation and additive genetic variance. A) The partial correlation between local adaptation and local environmental heterogeneity, controlling for additive genetic variance. B) The partial correlation between additive genetic variance and environmental heterogeneity, controlling for local adaptation. Statistical significance was assessed after correcting for multiple comparisons. The solid black line indicates the statistical null expectation of 0.

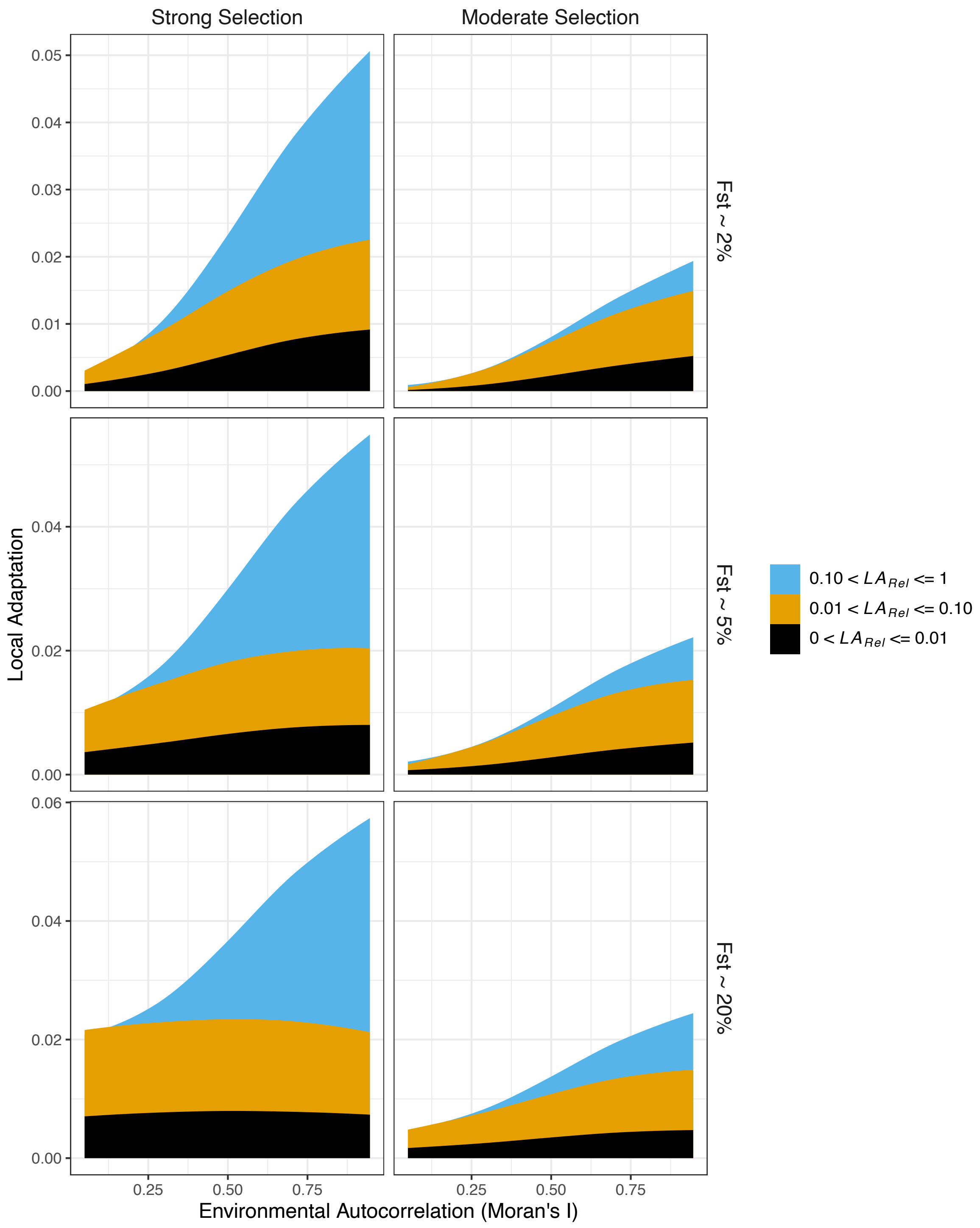

Supplementary Figure 8 The distribution of locally adaptive effects as a function of spatial autocorrelation in the environment. The area shown was calculated across 200 independent simulations and smoothed using a LOESS regression with span 1.5. The upper right cell is included in Figure 2 of the main text.

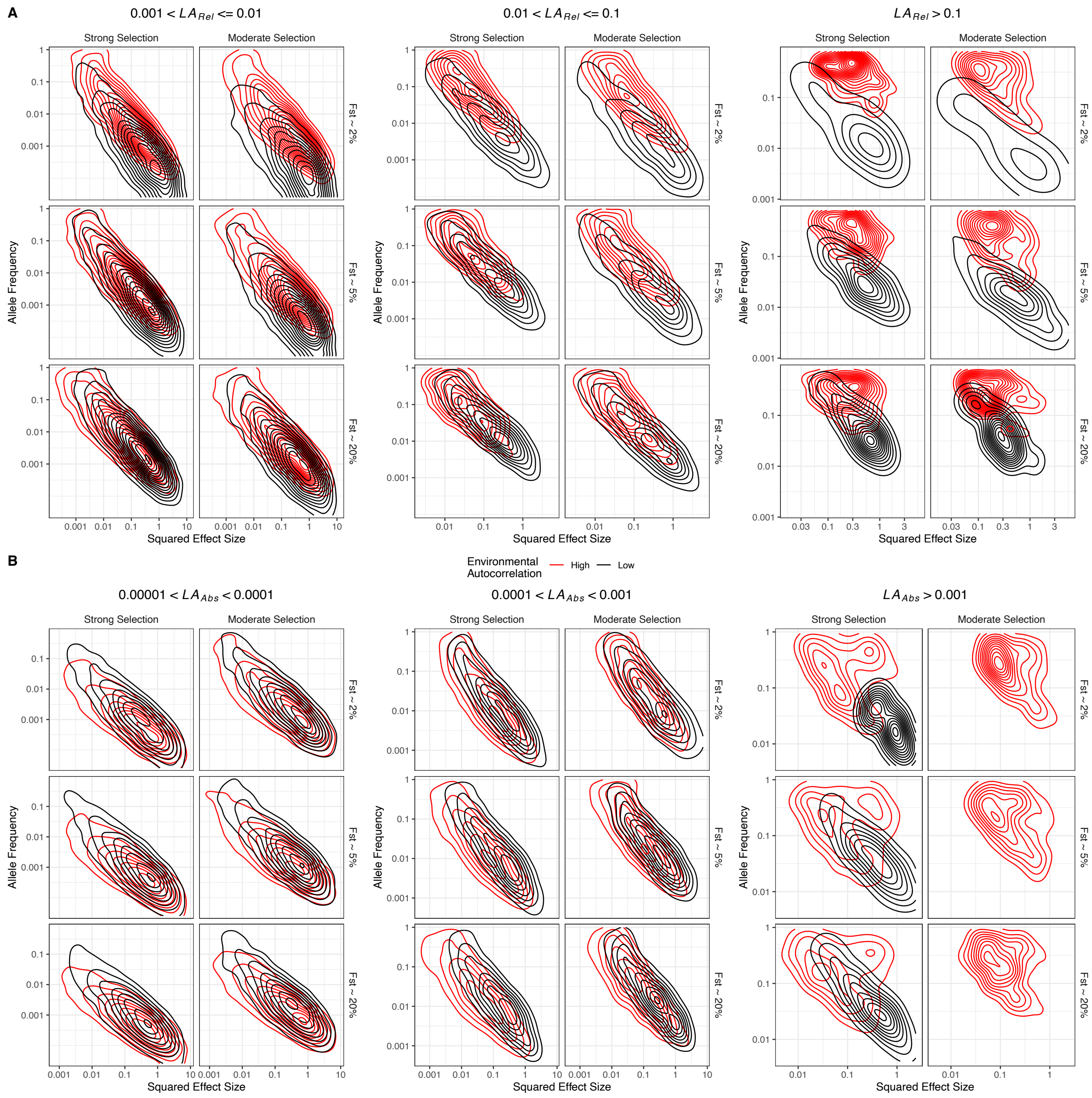

Supplementary Figure 9 The relationship between allele frequency and the squared phenotypic effect size for polymorphisms that contribute varying degrees of local adaptation in either relative (panel A) or absolute terms (panel B).

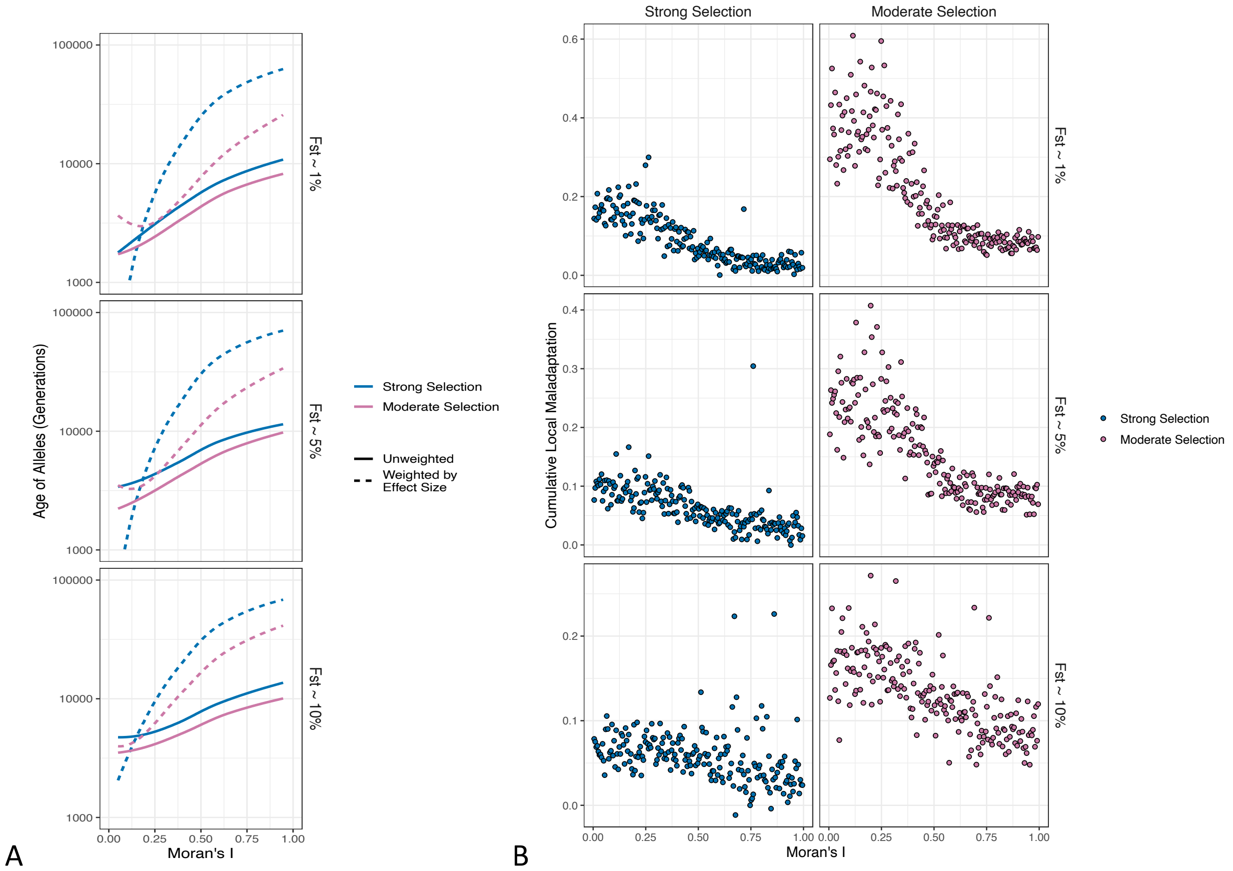

Supplementary Figure 10 A) Cumulative local maladaptation as a function of spatial autocorrelation in the environment across all parameter combinations. B) The average age of locally adaptive alleles in meta-populations subject to spatially varying selection. The lines represent LOESS regression curves calculated with span parameters of 1.5.

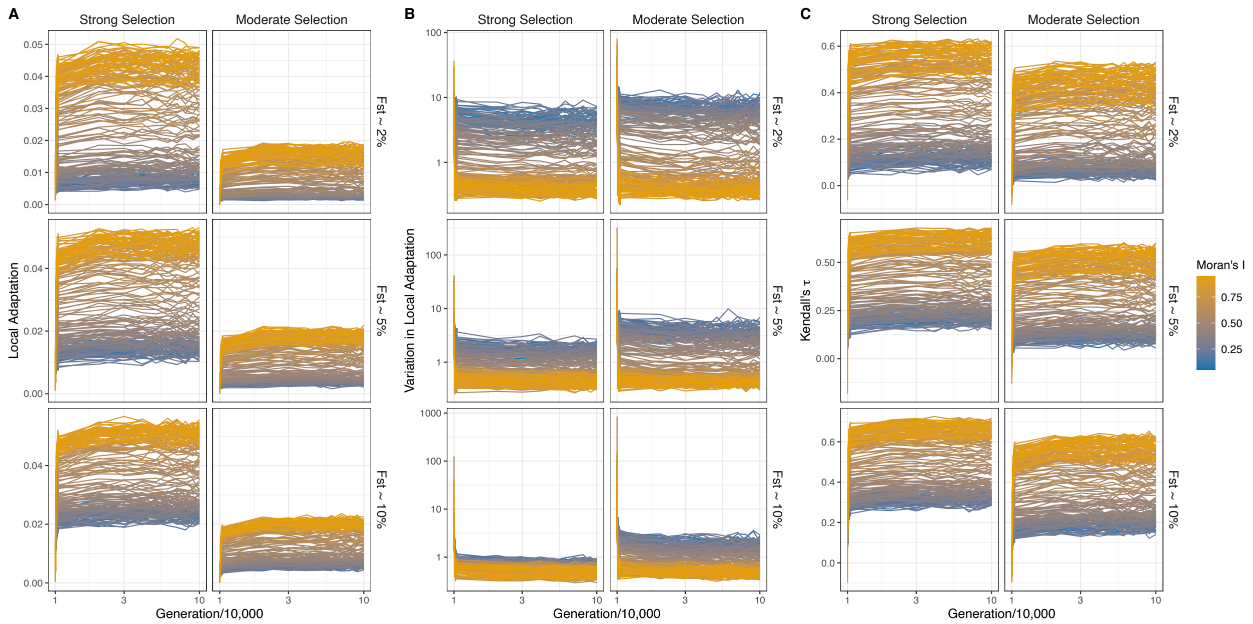

Supplementary Figure 11 Establishment of local adaptation in the simulations. Panel A) shows the average level of local adaptation across all demes. Panel B) shows the coefficient of variation in local adaptation across demes. Panel C) shows the Kendall's tau rank correlation between phenotypes and local optima.
